# Supplementary material for: Assessing biomass and primary production of microphytobenthos in depositional coastal systems using spectral information
Source: PLoS One. 2021 Jul 6;16(7):e0246012. doi: 10.1371/journal.pone.0246012 (PMC8259957; doi:10.1371/journal.pone.0246012)
Supplement: S1 File — Linear relationships between daily benthic primary productivity (mg C m-2 d-1) and corrected benthic chlorophyll-a concentrations (mg m-2) for three models with respect to vertical distribution of benthic algae in the top layer of the sediment (n = 6). (DOCX) [file pone.0246012.s001.docx]

**Supplement 1**

*Linear relationships between daily benthic primary productivity (mg C m^-2^ d^-1^) and corrected benthic chlorophyll-a concentrations (mg m^-2^) for three models with respect to vertical distribution of benthic algae in the top layer of the sediment (n=6).*

| **Distribution model** | **a** | **b** | **R^2^** | **p** |
| --- | --- | --- | --- | --- |
| Model 1 | -160.38 ± 238.92 | 2.17 ± 1.83 | 0.26 | 0.30 |
| Model 2 | -159.38 ± 225.73 | 2.10 ± 1.73 | 0.27 | 0.29 |
| Model 3 | -93.43 ± 159.31 | 1.35 ± 1.22 | 0.23 | 0.33 |
