# Supplementary material for: Assessing biomass and primary production of microphytobenthos in depositional coastal systems using spectral information
Source: PLoS One. 2021 Jul 6;16(7):e0246012. doi: 10.1371/journal.pone.0246012 (PMC8259957; doi:10.1371/journal.pone.0246012)

**Supplement 2**

*Correlations between photosynthetic parameters (being α^β^ as the slope of the light-limited part of the curve in mg C (mg CHLa)^-1^ ^h-1^ (PAR µE ^m-2 s-1^) ^-1^) and P^β^_max_ as the maximum photosynthetic production rate in mg C (mg CHLa ^-1^) h^‐1^) and environmental conditions (uncorrected chlorophyll-a, corrected chlorophyll-a and pheophytin-a concentrations (mg m^-2^), median grain size (µm) and mud percentage of the sediment) at the mudflats in the Ems estuary in September 2018, April 2019 and July 2019 (pooled data, n=6).*
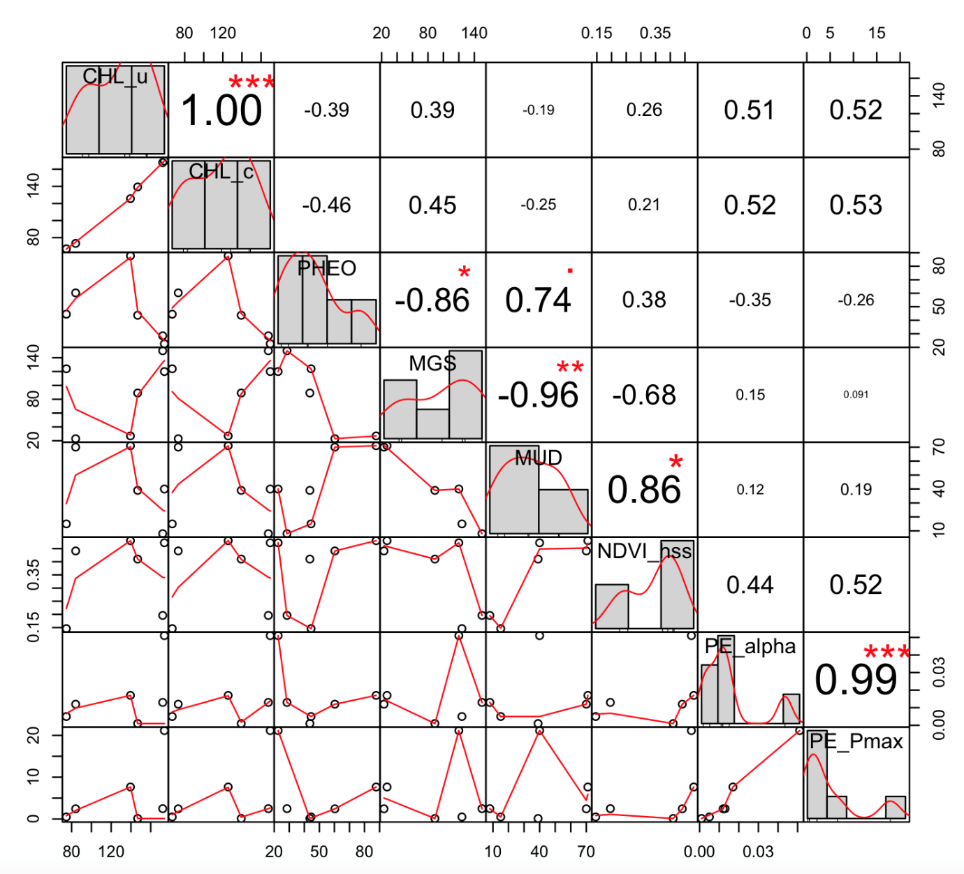

Supplement: S2 File — Correlations between photosynthetic parameters (being αβ as the slope of the light-limited part of the curve in mg C (mg CHLa)-1 h-1 (PAR μE m-2 s-1) -1) and Pβmax as the maximum photosynthetic production rate in mg C (mg CHLa -1) h−1) and environmental conditions (uncorrected chlorophyll-a, corrected chlorophyll-a and pheophytin-a concentrations (mg m-2), median grain size (μm) and mud percentage of the sediment) at the mudflats in the Ems estuary in September 2018, April 2019 and July 2019 (pooled data, n = 6). (DOCX) [file pone.0246012.s002.docx]
