# Supplementary material for: Assessing biomass and primary production of microphytobenthos in depositional coastal systems using spectral information
Source: PLoS One. 2021 Jul 6;16(7):e0246012. doi: 10.1371/journal.pone.0246012 (PMC8259957; doi:10.1371/journal.pone.0246012)
Supplement: S3 File — Linear relationships between corrected and uncorrected chlorophyll-a concentrations (mg m-2) as determined at the three sampling campaigns. (DOCX) [file pone.0246012.s003.docx]

**Supplement 3**

*Linear relationships between corrected and uncorrected chlorophyll-a concentrations (mg m^-2^)* as determined at the three sampling campaigns.

| Campaign | a | b | n | r^2^ | p |
| --- | --- | --- | --- | --- | --- |
| Sept 2018 | -16.32 ± 1.83 | 1.03 ± 0.01 | 15 | 1.00 | < 0.001 |
| April 2019 | -6.19 ± 1.65 | 1.01 ± 0.01 | 12 | 1.00 | < 0.001 |
| July 2019 | -8.73 ± 3.27 | 1.01 ± 0.03 | 9 | 1.00 | < 0.001 |
