# Supplementary material for: Assessing biomass and primary production of microphytobenthos in depositional coastal systems using spectral information
Source: PLoS One. 2021 Jul 6;16(7):e0246012. doi: 10.1371/journal.pone.0246012 (PMC8259957; doi:10.1371/journal.pone.0246012)

**Supplement 4**

*Relationships between NDVI* as determined by means of hyperspectral sensors (NDVI_hss; -) during the field surveys and derived from these data for red and near-infrared (NIR) of spectral bands of the Landsat 7 ETM (NDVI_L7;-), the Landsat 8 OLCI (NDVI_L8;-) and the Sentinel 2 (NDVI_S2;-). The different symbols indicate different periods (square: September 2018, circle: April 2019, triangle: July 2019), the diagonal line depicts a 1:1 relationship.


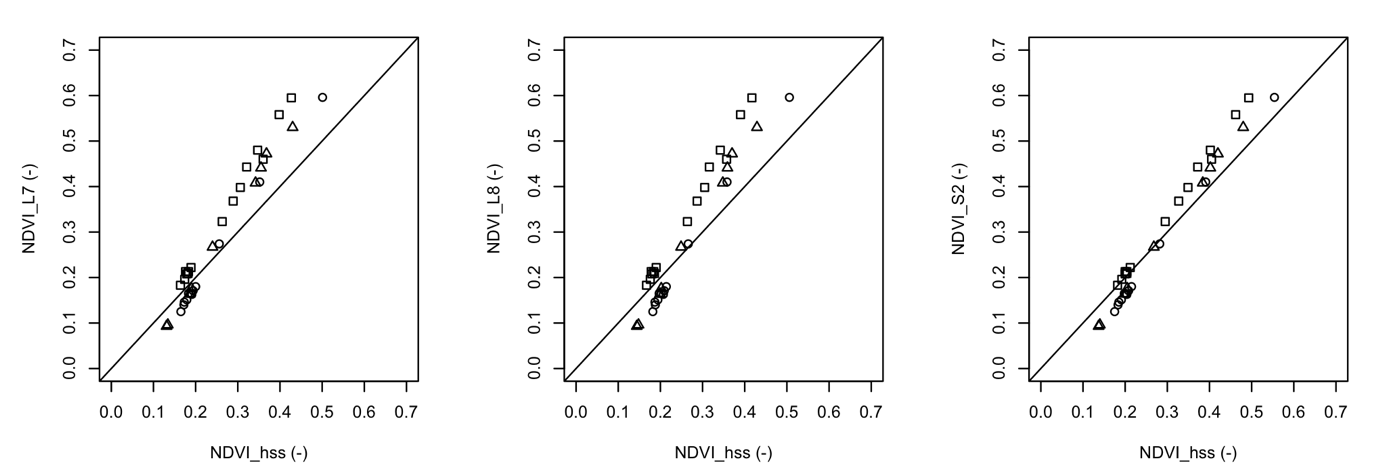

Supplement: S4 File — Relationships between NDVI as determined by means of hyperspectral sensors (NDVI_hss; -) during the field surveys and derived from these data for red and near-infrared (NIR) of spectral bands of the Landsat 7 ETM (NDVI_L7;-), the Landsat 8 OLCI (NDVI_L8;-) and the Sentinel 2 (NDVI_S2;-). The different symbols indicate different periods (square: September 2018, circle: April 2019, triangle: July 2019), the diagonal line depicts a 1:1 relationship. (DOCX) [file pone.0246012.s004.docx]
