# Supplementary material for: Assessing biomass and primary production of microphytobenthos in depositional coastal systems using spectral information
Source: PLoS One. 2021 Jul 6;16(7):e0246012. doi: 10.1371/journal.pone.0246012 (PMC8259957; doi:10.1371/journal.pone.0246012)
Supplement: S5 File — Sources: (1) DIAS ONDA: https://www.onda-dias.eu/cms/ (Level 1), and (2) Copernicus Open Access Hub: https://scihub.copernicus.eu/dhus (Level 2). (DOCX) [file pone.0246012.s005.docx]

**Supplement 5**

*Names of the Sentinel 2 images (tiles) downloaded. Sources: (1) DIAS ONDA:* [*https://www.onda-dias.eu/cms/*](https://www.onda-dias.eu/cms/) *(Level 1), and (2) Copernicus Open Access Hub:* [*https://scihub.copernicus.eu/dhus*](https://scihub.copernicus.eu/dhus) *(Level 2).*

| **Image name** | **Source** |
| --- | --- |
| S2A_MSIL1C_20180108T104421_N0206_R008_T32ULE_20180108T124506 | 1 |
| S2A_MSIL1C_20180108T104421_N0206_R008_T32ULE_20180108T124506 | 1 |
| S2A_MSIL2A_20180421T105031_N0207_R051_T32ULE_20180421T111316 | 2 |
| S2A_MSIL2A_20180508T104031_N0207_R008_T32ULE_20180508T175127 | 2 |
| S2B_MSIL2A_20180523T104019_N0208_R008_T32ULE_20180524T145942 | 2 |
| S2A_MSIL2A_20180607T104021_N0208_R008_T32ULE_20180607T132721 | 2 |
| S2A_MSIL2A_20180806T104021_N0208_R008_T32ULE_20180806T142805 | 2 |
| S2A_MSIL2A_20180918T105021_N0208_R051_T32ULE_20180918T141223 | 2 |
| S2A_MSIL2A_20181117T105321_N0210_R051_T32ULE_20181117T121932 | 2 |
| S2A_MSIL2A_20190215T105131_N0211_R051_T32ULE_20190215T120504 | 2 |
| S2B_MSIL2A_20190227T104019_N0211_R008_T32ULE_20190227T165024 | 2 |
| S2A_MSIL2A_20190625T105031_N0212_R051_T32ULE_20190625T134744 | 2 |
| S2B_MSIL2A_20190727T104029_N0213_R008_T32ULE_20190727T134640 | 2 |
| S2B_MSIL2A_20190826T104029_N0213_R008_T32ULE_20190826T140844 | 2 |
| S2B_MSIL2A_20200205T105129_N0214_R051_T32ULE_20200205T114124 | 2 |
