# Supplementary material for: Assessing biomass and primary production of microphytobenthos in depositional coastal systems using spectral information
Source: PLoS One. 2021 Jul 6;16(7):e0246012. doi: 10.1371/journal.pone.0246012 (PMC8259957; doi:10.1371/journal.pone.0246012)
Supplement: S6 File — Images for the Dollard at which the tidal flats were fully exposed in 2018 and 2019. Astronomical low tide is given for Nieuwe Statenzijl (bordering the Dollard estuary in the south). The timing of the satellite image of 18 September 2018 coincided with that of the 1st sampling cruise and whilst that of 27th July 2019 was taken three days after the 3rd sampling cruise. (DOCX) [file pone.0246012.s006.docx]

**Supplement 6**

*Overview of cloudless Sentinel 2 images of the Dollard at which the tidal flats were fully exposed in 2018 and 2019. Astronomical low tide is given for Nieuwe Statenzijl (bordering the Dollard estuary in the south). The timing of the satellite image of 18 September 2018 coincided with that of the 1^st^ sampling cruise and whilst that of 27^th^ July 2019 was taken three days after the 3^rd^ sampling cruise.*

| **Satellite** | | | **Low tide** | | **Timing** |
| --- | --- | --- | --- | --- | --- |
| **Date** | **Day nr** | **UTC** | **Local time** | **UTC** | **ST compared to LT** |
| 8 January 2018 | 8 | 10:44 | 11:33 | 10:33 | Around LT |
| 7 February 2018 | 38 | 10:42 | 11:36 | 10:36 | Around LT |
| 21 April 2018 | 111 | 10:50 | *11:43* | 09:43 | 1 hr before LT |
| 08 May 2018 | 128 | 10:40 | *12:31* | 10:31 | Around LT |
| 23 May 2018 | 143 | 10:40 | 13:48 | 11:48 | 1 hr after LT |
| 07 June 2018 | 158 | 10:40 | 13:02 | 11:02 | Around LT |
| 06 August 2018 | 218 | 10:40 | 13:59 | 11:59 | Around LT |
| 18 September 2018 | 261 | 10:50 | 13:10 | 11:10 | Around LT |
| 17 November 2018 | 321 | 10:53 | 12:46 | 11:46 | 1 hr after LT |
| 15 February 2019 | 46 | 10:51 | 13:56 | 12:56 | 2 hrs after LT |
| 27 February 2019 | 58 | 10:40 | 12:15 | 11:15 | Around LT |
| 25 June 2019 | 176 | 10:50 | *12:29* | 10:29 | Around LT |
| 27 July 2019 | 208 | 10:40 | *14:28* | 12:28 | 2 hrs after LT |
| 26 August 2019 | 238 | 10:40 | *14:55* | 12:55 | 2 hrs after LT |
| 5 February 2020 | 36 | 14:12 | 14:35 | 13:35 | Around LT |
