# Supplementary material for: Assessing biomass and primary production of microphytobenthos in depositional coastal systems using spectral information
Source: PLoS One. 2021 Jul 6;16(7):e0246012. doi: 10.1371/journal.pone.0246012 (PMC8259957; doi:10.1371/journal.pone.0246012)
Supplement: S7 File — Linear relationships between corrected benthic pigment concentrations as a function of NDVI as determined by means of hyperspectral sensors (NDVI_hss; -) and those derived from these data for red and near-infrared (NIR) of spectral bands of the Landsat 7 ETM (NDVI_L7;-), the Landsat 8 OLCI (NDVI_L8;-) and the Sentinel 2 (NDVI_S2;-) during the field surveys in April and July 2019, with benthic pigments as corrected chlorophyll-a concentrations (CHLa_c), the sum of corrected chlorophyll-a and pheophytin-a concentrations (CHPH_c) and as uncorrected chlorophyll-a concentrations (CHLa_u) (n = 20). (DOCX) [file pone.0246012.s007.docx]

**Supplement 7**

*Linear relationships between corrected benthic pigment concentrations as a function of NDVI* as determined by means of hyperspectral sensors (NDVI_hss; -) and those derived from these data for red and near-infrared (NIR) of spectral bands of the Landsat 7 ETM (NDVI_L7;-), the Landsat 8 OLCI (NDVI_L8;-) and the Sentinel 2 (NDVI_S2;-) during the field surveys in April and July 2019, with benthic pigments as corrected chlorophyll-a concentrations (CHLa_c), the sum of corrected chlorophyll-a and pheophytin-a concentrations (CHPH_c) and as uncorrected chlorophyll-a concentrations (CHLa_u) (n=20).

| **Sensor** | **intercept** | **slope** | **r^2^** | **p** |
| --- | --- | --- | --- | --- |
| **CHLa_c** |  |  |  |  |
| Hyperspectral sensors | 63.7 ± 13.1 | 165.1 ± 44.3 | 0.42 | 0.001 |
| Landsat7 | 44.3 ± 17.1 | 251.2 ± 64.8 | 0.44 | 0.001 |
| Landsat8 | 38.4 ± 18.5 | 263.2 ± 67.7 | 0.44 | < 0.001 |
| Sentinel2 | 47.8 ± 16.5 | 215.8 ± 56.5 | 0.44 | 0.001 |
| **CHPH_c** |  |  |  |  |
| Hyperspectral sensors | 93.1 ± 11.3 | 186.8 ± 38.5 | 0.55 | < 0.001 |
| Landsat7 | 71.7 ± 14.9 | 281.6 ± 56.3 | 0.57 | < 0.001 |
| Landsat8 | 65.6 ± 16.2 | 293.7 ± 59.2 | 0.56 | < 0.001 |
| Sentinel2 | 75.6 ± 14.4 | 242.4 ± 49.2 | 0.56 | < 0.001 |
| **CHLa_u** |  |  |  |  |
| Hyperspectral sensors | 68.8 ± 12.5 | 169.1 ± 42.4 | 0.46 | < 0.001 |
| Landsat7 | 49.0 ± 16.4 | 256.8 ± 62.0 | 0.47 | < 0.001 |
| Landsat8 | 43.1 ± 17.7 | 268.8 ± 64.9 | 0.47 | < 0.001 |
| Sentinel2 | 52.6 ± 15.8 | 220.7 ± 54.1 | 0.47 | < 0.001 |
