# Supplementary material for: Assessing biomass and primary production of microphytobenthos in depositional coastal systems using spectral information
Source: PLoS One. 2021 Jul 6;16(7):e0246012. doi: 10.1371/journal.pone.0246012 (PMC8259957; doi:10.1371/journal.pone.0246012)
Supplement: S8 File — Two sampling stations in 2018, one located within the study area (Groote Gat Noord) and the other one just northwest of this (Bocht van Watum). A) Sea surface temperature (° Celcius), with the green line indicating the freezing point of freshwater (0°C). B) Salinity (psu). C) Concentrations of phosphate (PO42-; mg l-1. D) Concentrations of ammonia (NH4+; mg l-1). E) Concentrations of the sum of nitrite and nitrate (NO22-; + NO32-; mg l-1). Data source: RWS. The grey vertical lines indicate the sampling dates of the satellite images, the blue line that of the field survey in 2018. (DOCX) [file pone.0246012.s008.docx]

**Supplement 8**

Seasonal dynamics in environmental conditions at two sampling stations in 2018, one located within the study area (Groote Gat Noord) and the other one just northwest of this (Bocht van Watum). A) Sea surface temperature (° Celcius), with the green line indicating the freezing point of freshwater (0°C). B) Salinity (psu). C) Concentrations of phosphate (PO_4_^2-^; mg l^-1^. D) Concentrations of ammonia (NH_4_^+^; mg l^-1^). E) Concentrations of the sum of nitrite and nitrate (NO_2_^2-^; + NO_3_^2-^; mg l^-1^). Data source: RWS. The grey vertical lines indicate the sampling dates of the satellite images, the blue line that of the field survey in 2018.


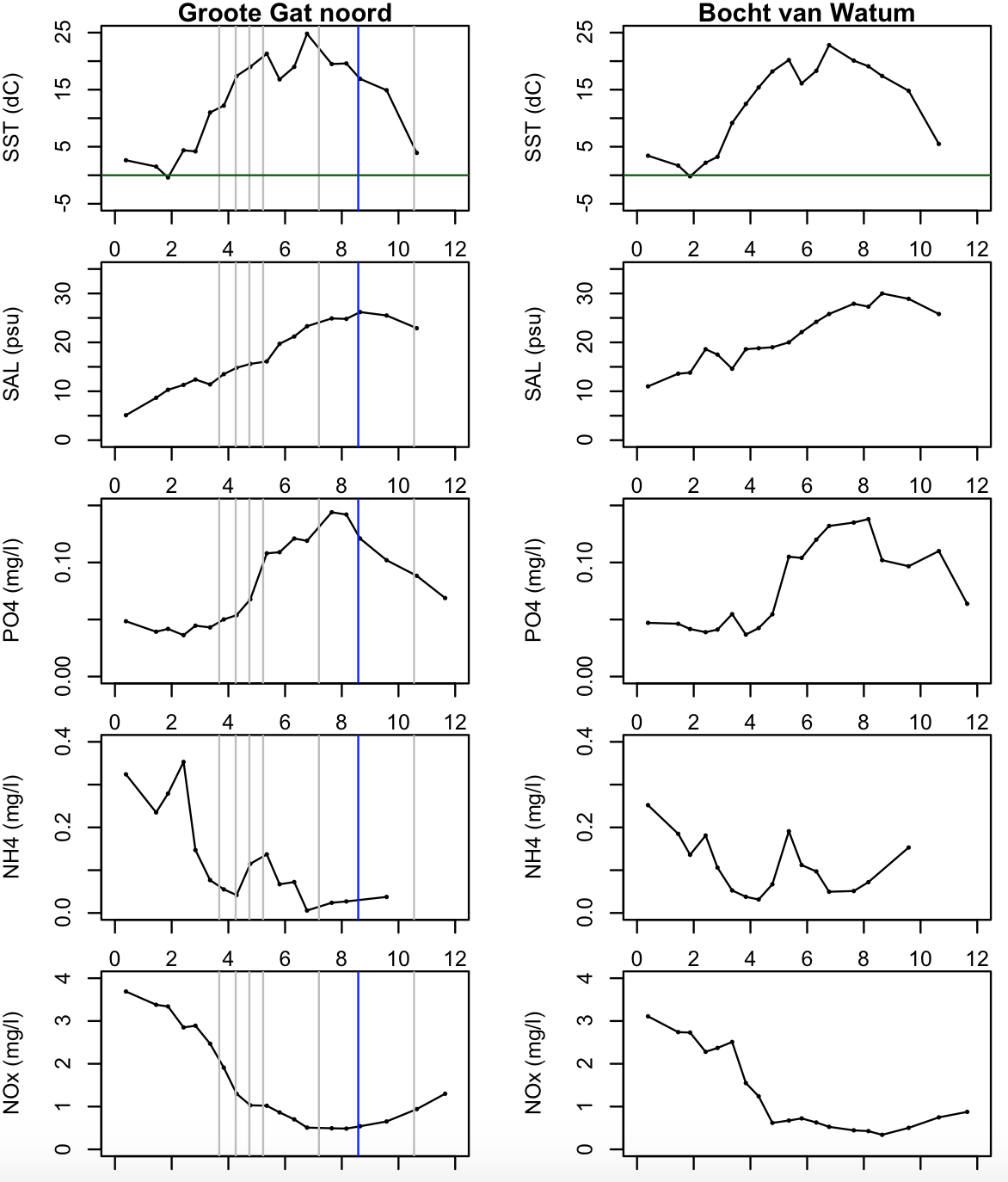


**A**

**B**

**C**

**D**

**E**
