# Supplementary material for: Assessing biomass and primary production of microphytobenthos in depositional coastal systems using spectral information
Source: PLoS One. 2021 Jul 6;16(7):e0246012. doi: 10.1371/journal.pone.0246012 (PMC8259957; doi:10.1371/journal.pone.0246012)
Supplement: Attachment — Submitted filename: Response to reviewers.docx [file pone.0246012.s010.docx]

Dear editor,

We were pleased to hear that you think our manuscript has merit and we would like to thank you and the two reviewers for their constructive comments on our manuscript “Assessing biomass and primary production of microphytobenthos in depositional coastal systems using spectral information.” [Jacobs et al. PONE-D-21-01011].

Rodney Forster remarked that it is a good manuscript, with a solid data set and that the satellite data adds an extra dimension to help understand coastal systems. His main comment regards the PAR values used. He noted a mistake in the conversion from Watt to microEinstein. We thank the reviewer for addressing this mistake! This has been corrected in this revised version.

Dominique Davoult thinks that the data set presented is interesting and of quality. He comments that methodological flaws should be discussed in more detail, which we believe we have done this now in this revised version.

Below is our point-by-point reply to the comments of both reviewers.

Based on the comments and suggestions made, we think the manuscript has been greatly improved. We appreciated that the reviewers made their identity known to us. We would like to include their names in the acknowledgements but would like to check with you and the reviewers if this is appropriate.

We hope that you will consider the revised manuscript for publication in PLOS ONE.

With kind regards,

Pascalle Jacobs on behalf of all authors.

Review comments PLOS ONE

“Assessing biomass and primary production of microphytobenthos in depositional coastal systems using spectral information.” Jacobs et al. [PONE-D-21-01011]

***Reviewer #1: Rodney Forster***

It is a good paper and brings another solid data set to the calculation of estuarine productivity. Using satellite data adds another dimension to our ability to understand these systems. There are plausible explanations of why biomass (and productivity??) are low in the summer for tidal flats. My feeling is that this is due to grazing being higher than production through the summer.

Please see my comments on the paper marked on the pdf version attached. It requires some changes before acceptance, chiefly the PAR values from the met station are too high. Correction to these will also require correction of the daily primary production as they are used in modelling. I look forward to seeing a revised version, and eventual publication.

The reviewer made additional comments in the manuscript:

1.	p3, L30: Text suggestion to change sentence

*-Suggestion adopted.*

2.	p3, L50: Text suggestion (typo)

*-Changed “interlined” into “interlinked”.*

3.	p4, L74: suggestion to address gap of including mpb pp in monitoring programs in discussion

*-Added to paragraph 4.5*

4.	p6, L126: Replace silt by mud

*- Suggestion adopted.*

5.	p6, L139: text suggestion: “man-induced” into “human-induced”

*- Suggestion adopted.*

6.	p7, L150: “Can you say more about the two primary production stations? Were they at different shore heights, or close together? Can their positions be indicated on the map if different from the chlorophyll stations?”

*-Chlorophyll-a samples were taken at several stations, depending on sampling date, for two of these stations, samples were also obtained for 14C incubations. This has been added to the text. In tables 2 and 3 the PrimProd stations are indicated by shaded cells. The GPS-coordinates have been added to Table 2 (sediment) and they are referred to in the caption of Table 3 (chlorophyll).*

7.	p7, L152: Were sediment samples taken from the same depth as chla samples?

*-Yes, this information now added to the M&M section.*

8.	p9, L199: What was the temperature inside the photosynthetron?

*-Temperatures were kept at in-situ water temperatures as measured during sampling. This is added.*

9.	p10, L233: There is a mistake in the conversion of light‼! Check, recalculate, redo the analysis and check the discussion after new analysis.

*-Thank you for pointing this out. Conversions from Watt to µEinstein was done by multiplying by 4.66 and then multiplied by 0.45 to obtain the PAR spectrum. The underlined section somehow got deleted in the text. The daily production rates were calculated with the correct PAR data (see data supplied in DOI).*

*When constructing Figure 7, we did used the incomplete/incorrect conversion from Watt to µEinstein. A new figure 7 has been constructed with the correct irradiance values (multiplied by 0.45 to obtain PAR).*

10.	p12, L287: “RAMSES-ARC-VIS, MMS-1 monolithic diode array mini spectrophotometer” Comment: I think the RAMSES contains a MMS-1 spectrometer.

*-In Table 1 we have removed MMS-1 from the column ‘sensor’ and added RAMSES for paper [35].*

11.	p12, Table 1: two text suggestions (Easter and Wester should both have an “n”)

*-This has been corrected.*

12.	p13, L313: Replaced ‘to’ by ‘on the’.

*- This has been corrected.*

13.	p17, L400: wrong numbers due to wrong par: redo.

*-Please see reply to comment nr. 9, PAR used to construct curves and daily production was correct, except that in the conversion there was a slight rounding error which has now been corrected. This resulted in slightly higher daily production rates.*

14.	p17, L401: use air temperatures not water.

-Air temperatures have been added to Table 4 and these are now added to paragraph 3.2.

15.	p17, L407: add as main message that Pb is vary variable.

*-This has been added to the text.*

16.	p18, Table 5: add emersion time to table if stations were of different heights.

*-Emersion time was different between dates, but not between the stations at each date. Times were added to Table 5.*

17.	p22, L515: Replace “as was found” by “in contrast to that found for”.

*-Change has been adopted.*

18.	p22, L519-523: “see results of Kromkamp et al 2020 in Frontiers MPB special issue for high temporal resolution changes in MPB surface reflectance on undisturbed biofilms. Large changes in NDVI for essentially the same chlorophyll biomass.”

*-We thank the reviewer for the suggestion, the reference to the paper has been added.*

19.	p22, L526: “an alternative approach would be to try and correct for vertical migration using the tide- and sun-angle calculations of Pinckney & Zingmark. Kromkamp et al 2020 above showed a fair degree of success using this method for Westerschelde MPB.”

*-We thank the reviewer for this suggestion, and we have done the calculation as suggested*

*Unfortunately, this did not result in a better relation between NDVI and biomass.*

20. p22, L529: units mg m-2 per NDVI interval

*-Correct unit added to the text.*

21. p22, L531: “what does this mean for the Ems Dollard? Is there more sub-surface chlorophyll present which does not contribute to NDVI?”

*-We think that this is indeed the case, this clarification has been added to the manuscript.*

22. p22, L534: “bear in mind here that Phaeopigments have a much higher blue than red absorption peak. If you analyse the hyperspectral curves, you will see that there are shifts in the blue region 400-435 nm which reflect the degradation of chl to phaeo.”

*-We thank the reviewer for this addition. In general, we have added some extra sentences to the discussion stressing more insight in the contribution of other pigments (see reply to Q29).*

23. p23, L552: “irradiance is rarely higher than 1600 at noon at this latitude, only on cloud-free days. The average monthly irradiance during tidal emersion is the number to consider here, not the maximum.”

*-We agree with the reviewer, the sentence has been rewritten and we are no longer using Eopt, only Ek.*

24. p23, L558-559: “Eopt mean what it says, optimal for photosynthesis. Does not mean that levels below this are too low for growth. Growth in diatoms is possible at very low light levels.”

*-We agree with the reviewer we are no longer using Eopt, only Ek.*

25. p23, L561: “How were the thermal tolerance values developed?”

*-A new Figure 7 has been constructed, with thermo-inhibition now at 25C. This value has been taken from literature (Blanchard et al., 1997; Morris & Kromkamp, 2003).*

26. p24, L571: “To improve this part - look for the 'marine heatwave' events of 2018 and 2019, the weeks when air temperature is consistently above 25°C for a prolonged period. Mark these short, but important periods, on the Figures and link to the growing literature on marine heatwave events. Mention that temperatures at the mud surface can be much warmer than the air.”

*-We thank the reviewer for the suggestion. In Figure 7 the threshold for thermo-limitation is placed at 25 C, and in the text, we discuss the possibility of a reduced production in summer by relating this to the number of days the temperature was >25C.*

27. p24, L585: “resuspension of MPB” the reviewer remarks that the study area is in enclosed bay.

*-This is added to the text.*

28. p25, L599: “frequency of images can be higher if moderate resolution sensors such as Sentinel-3 are used. This was done to great effect by van der Wal et al. in their multi-estuary comparison of seasonality.”

-*That is correct, and it can certainly be done. However, for an environment with a very patchy environment such the mudflats in the Ems Dollard, a high spatial resolution is much preferred, so Sentinel 2 at 10 m is the choice. This is particularly true in matchup analysis, because point in-situ measurements have to be associated to a pixel that has to be as small as possible. For regular monitoring, Sentinel 3 could be used, although given the size and geographic characteristics of the area, much of its surface would either be left out or subject to pixel contamination.*

29. p25, L613: “Key to resolve this would be to use HPLC to look for phytoplankton pigment tracers e.g. 19-hex, or chl-b which are not found in MPB.”

*-We thank the reviewer for this addition, the suggestion to use HPLC has been added to the manuscript.*

30. p25, L619-620: “Values seem unrealistically high. I have doubts about the PAR values used here. The surface values from the met station are two time too high. Can the authors double check on the measurements with the Walz sensor in their photosynthetron? Measure again with a calibrated Li-cor sensor to check.”

*-As part of a long-term series 14C measurements are performed at our institute 40x a year to obtain pelagic production. On each occasion, light inside the vials in the photosyntheron are measured. I checked the light intensities measured done for the sample dates around the dates our benthic measurements took place. The given intensities seem to be correct. In addition, the sensor was calibrated just before the start of this project, in July 2018, by the manufacturer.*

31. p25, L622: “It is very difficult to get more than 1000 µmol m-2 s-1 in a photosynthetron set up without generating a lot of heat.”

*-We reach these high intensities by using halogen light bulbs, the water inside the incubator is cooled and kept at the set in-situ water temperature. We check temperature during the two-hour incubation. The range of light intensities was between 65 and 1522* µ*E m-2 s-1 (not 1825) for the 3 incubations. This is added to the text.*

“Diatoms generally light saturate at 300-800 µmol.”

*-Eopt is removed from Table 4 and references to this parameter are removed throughout the text. The estimates were from the model came with large standard errors and values were beyond intensities used in the incubator. In Figure 7 an estimate is made on PAR values above which photoreduction might take place using values from literature.*

32. p25, L623: “yes, they were in range, but show a tremendous variability.”

*-The large variability between stations and samples dates is added to the text.*

33. p25, L624: “these very low values in April need explanation. It is near the peak of the spring bloom, so we know that the biofilm is growing. Cells were resuspended in filtered seawater, so no carbon or nutrient limitation. Why such low Pbmax? Were any PAM measurements done? Was fv/fm also low?”

-*No other measured have been performed, so unfortunately, we do not have any independent method to compare the results. Production was low in relation to the chlorophyll-a concentrations, mainly due to a low P^b^max. When looking at the air temperatures, it was observed that on and a few days before the sampling date in April,* *minimum temperatures at 10cm above the ground were below zero (-2.7C). this cold spell occurred after a mild period, with air temperature being >20 C (www.knmi.nl). It is known that low temperatures have an impact on P^b^max (Morris & Kromkamp, 2003), and the only plausible explanation we can think of is a sudden drop in Pmax, resulting low production caused by this cold spell. This has been added to the text.*

*The sentence is replaced by “**The low values for αβ and Pβmax found in April (Table 4) might have been the result of low temperatures as will be discussed below.”*

34. p28, L705-706: “I don't think that is a valid comparison. The exact stations of the 1970s would have to be revisited, and the same methods used. They probably did not take a 2mm surface sample at that time, more likely to be a 1 cm core.

*-We agree with the reviewer and the sentence is removed from the text.*

35. p29, L714” …it could also imply that this relationship is no longer true. “Important point, relate to the marine heatwave literature as many people are now working on these extreme events.”

*-The suggestion is adopted.*

36. p29, L717-719: remove lines with comment:” redo the analysis with correct PAR values.”

*-The analyses were done with the correct PAR values (see also reply to comment nr.9).*

37. p29, 719 onwards: “The three field campaigns are not enough to constrain annual variability in PP. Why not use the satellite data to establish the photosynthetic biomass, and improve the estimate?”

*-We agree with the reviewer, therefore we have made an estimation of annual variability using the satellite date and this estimate was added to the text.*

**Reviewer #2: Dominique Davoult**

The questions discussed in the paper are very interesting but complex. Several previous publications have already discussed these points and concluded about the difficulty to assess biomass and mainly production using spectral information. The data set presented her is interesting and seems to be of quality. I consider that the manuscript could deserve publication after addressing some important points.

The authors highlighted important questions but did not really solve them.

1. First of all, they pointed out an important flaw, that is the fact that satellites measures allow an accurate estimation of Chl.a + Pheophytin-a but not of chl.a alone, that is a major problem to use these data to estimate productivity from chl.a estimates and light availability.

*- We agree with the reviewer that we consider this a flaw which deserves more attention (in particular when biomass is converted to productivity). We see (at least) two possible solutions.* *First, more attention should be given to develop algorithms that accurately estimate chlorophyll-a (and not the sum of the pigments chl-a and pheo-a). Second, if this is not possible then the fraction of chl-a of the total pigment concentration should be derived from ground truthing (in situ field observations). We have now added these suggestions to the ‘discussion’ section of the manuscript.*

2. Another flaw is the way the authors measured productivity: they used a lab method, very precise but very far from the actual field productivity. 14C measurements are interesting for physiological considerations and produce potential production as a function of controlled variables but measurements are performed in very artificial conditions, very far from the real life. It can be one of the reasons of the poor correlation between satellite data and estimated productivity. It would be at least necessary to perform measurements on intact sediment samples or, better, to perform in situ measurements of benthic productivity: these two approaches are now quite common and certainly better estimations. The methods used here must be more critically discussed and justified.

*-We agree with the reviewer that we should justify the method used in this study and critically discuss the chosen methods as well as alternatives. This is now added to the text under ‘methodology’ in paragraph 4.2.*

3. Migne et al. (2007, J.Phycol.) tried to do the same kind of approach by using in situ fluorescence measurements to estimate microphytobenthic production. However, in a further paper, the same team (Davoult et al., 2009, Hydrobiologia) demonstrated that the relationship was strongly locality-dependent and should be used with caution despite they obtained a stronger explained variance than you. I believe you should discuss that point.

*-We agree with the reviewer that the relations described in our manuscript between NDVI and chlorophyll-a apply specifically to the area studied. This is now stated more explicitly in the discussion.*

Some particular points:

4.	p.6-L.128: salinity is now expressed without unit, like a density.

*-At out lab we measure salinity by reading the refraction index of 0.2 µm-filtered seawater, acclimatised to laboratory temperature, using a handheld refractometer (ENDECO type 102, accuracy 0.1‰). The refraction index (or salinity) is then corrected for temperature using temperature-salinity charts. The salinity is given as the practical salinity unit, abbreviated as psu, indeed with no unit.*

5.	p.16, L. 390: values of the ratio between pheophytin-a and the sum of corrected chl.a and pheophytin-a seem to be wrong and cannot be deduced from table 3, please check.

*- The ratio between pheo and the sum of chla+pheo: to obtain the fraction pheo of total (chl+pheo) the average fraction per period has been calculated and divided by the average sum. This has been clarified in the text. The number for September was incorrect (typo) 2 should be 26%, this has been corrected.*

*The numbers have been rounded to integers for better readability, the sum of corrected chl and pheo have been checked, numbers were corrected if needed. The sum “CHLPH_c” varied between 102 and 412 mg m-2, this can be read from Table 3 (min April 2019, station 6; max sept 2018 station 5).*

6.	p24, L576: OK but data [on macrofauna density] not from the same year, you should be more cautious because of the high spatial and temporal variability of such results.

-*We agree with the reviewer that the data is not very recent. Our colleagues that are responsible for this monitoring program have provided us with more recent data (NIOZ unpublished data). In the figure below for the Dollard area the average AFDW (g m-2) of sampling points lay between a minimum of 1.5 and 4.5 g m-2 for the period 2009-2019. The sentence in the text has been rewritten.*

7.	p25, L619-620: these "optimum" values are questionable (and very high!), it seems better to rely on Ek.

-*We agree with the reviewer, Eopt values were estimated by model exercise, st. error was high. Eopt is removed from table 4 and references in the text as well. In the new version of the manuscript only Ek is used.*

8.	p28, L 685-688: I agree with this remark, the same could be apply on the way you measure productivity!

*-We agree with the reviewer, we have discussed the problems surrounding measuring primary production more thoroughly in paragraph 4.2 based on the suggestions you made.*

**Journal requirements**

1. Please ensure that your manuscript meets PLOS ONE's style requirements, including those for file naming.

*-The revised manuscript should now meet the journals’ requirements regarding style.*

2. In your Methods section, please provide additional location information of the study sites, including geographic coordinates for the data set if available.

*-This has been taken care of, coordinates were added to Table 2.*

3. In your Methods section, please provide additional information regarding the permits you obtained for the work. Please ensure you have included the full name of the authority that approved the field site access and, if no permits were required, a brief statement explaining why.

*-Details regarding the permit have been added to the Methods section.*

4. We note that you have stated that you will provide repository information for your data at acceptance. Should your manuscript be accepted for publication, we will hold it until you provide the relevant accession numbers or DOIs necessary to access your data. If you wish to make changes to your Data Availability statement, please describe these changes in your cover letter and we will update your Data Availability statement to reflect the information you provide.

*-All data used in preparing this manuscript (except for open access data) have been made available in DOI 10.4121/13516259. At present the data is checked by the data repository (4TU.ResearchData) and after approval the DOI will be made active.*

5. We note that Figures 1 and 5 in your submission contain map/satellite images which may be copyrighted. All PLOS content is published under the Creative Commons Attribution License (CC BY 4.0), which means that the manuscript, images, and Supporting Information files will be freely available online, and any third party is permitted to access, download, copy, distribute, and use these materials in any way, even commercially, with proper attribution. For these reasons, we cannot publish previously copyrighted maps or satellite images created using proprietary data, such as Google software (Google Maps, Street View, and Earth). For more information, see our copyright guidelines: http://journals.plos.org/plosone/s/licenses-and-copyright.

We require you to either (1) present written permission from the copyright holder to publish these figures specifically under the CC BY 4.0 license, or (2) remove the figures from your submission:

*- The satellite images are not copyrighted. All images are made using Sentinel 2 data. In the caption with Figure 1 the source from the Copernicus programme (open policy) is named. This reference is now also added to the caption of Figure 5. In addition, in the acknowledgements we have added the sentence “We acknowledge the European Commission and the European Space Agency for the Sentinel-2 images.”*
